# How much do Latin American medical students know about radiology? Latin-American multicenter cross-sectional study

**DOI:** 10.1080/10872981.2023.2173044

**Published:** 2023-02-01

**Authors:** Juan S. Izquierdo-Condoy, Katherine Simbaña-Rivera, Humberto Alejandro Nati-Castillo, Arthur Cassa Macedo, Claudia Diana Cardozo Espínola, Gabriela M. Vidal Barazorda, Ideli Palazuelos-Guzmán, Brayan Trejo García, Sarah J. Carrington, Esteban Ortiz-Prado

**Affiliations:** a OneHealth Global Research Group, Universidad de las Américas, Quito, Ecuador; b Health Management and Research Area, Universidad Internacional Iberoamericana, Arecibo, Puerto Rico; c Facultad Ciencias de la Salud, Universidad del Quindío, Armenia Colombia; d Department of Neurology and Neurosurgery, McGill University, Montreal, Canada; e Faculdade de Medicina, Universidades Federal de Minas Gerais, Belo Horizonte, Brasil; f Internal Medicine Service, Hospital General de Luque, Luque, Paraguay; g Facultad de Ciencias de la Salud, Universidad Privada San Juan Bautista, Lima, Perú; h Facultad de Ciencias de la Salud, Universidad Franz Tamayo, La Paz, Bolivia; i Facultad de Medicina, Universidad Autónoma del Estado de México, Ciudad de México, México; j Department of Economics, Universidad de las Américas, Quito, Ecuador

**Keywords:** Knowledge, radiology, students, medical, academic training, Latin america, teaching methods

## Abstract

**Background:**

Radiology is a useful tool for diagnosis and intervention in medical practice, and all the components within the teaching-learning process of this subject during undergraduate studies influence successful knowledge application.

**Objective:**

This study aimed to describe the level of knowledge in radiology of students in the last two years of medical school and curricular characteristics of their courses in seven Latin American countries.

**Methods:**

A multicenter cross-sectional study was carried out on medical students of 7 Latin American countries (Bolivia, Brazil, Colombia, Ecuador, Mexico, Paraguay, and Peru) in their final two years of medical school, using an online questionnaire validated by experts and adapted for each country that assessed knowledge and curricular characteristics in radiology subject. Scores were assigned according to the number of correct answers for the knowledge test. The T-test, and regression analysis with one-way ANOVA were used to search for relationships between the level of knowledge and other variables.

**Results:**

A total of 1514 medical students participated in this study. All countries had similar participation (*n* > 200); most participants were women 57.8%. The country with the highest knowledge score was Brazil. Male, sixth year (internship) and from public universities students had higher knowledge score (*n* < 0.05). Participants, who considered radiology more important, and who reported higher compliance with teaching staff with the proposed syllabus, and programmed classes, obtained better scores (*n* < 0.05).

**Conclusions:**

Latin American medical students included in this study have a regular overall level of knowledge of Radiology, apparently influenced by curricular differences such as class and academic program compliance. Efforts to better understand and improve academic training are indispensable.

**Limitations:**

The study was subject to selection bias determined by non-probability convenience sampling. The questionnaire assessed only theoretical knowledge and the evaluation system was designed by the investigators.

## Introduction

Radiology debuted as a medical specialty at the end of the 19th century. Since then, imaging techniques have shown exponential advances, starting with studies of low complexity such as radiography, and reaching more complex techniques such as ultrasound, computed tomography, and magnetic resonance [1,2]. Nowadays, radiology represents a fundamental part of medical practice, assisting in the detection, evaluation of extension and complications of cancer, and treatment of many diseases [3,4]. However, this has not been reflected in the undergraduate curricula of medical schools [2].

The classic teaching model proposed by Abraham Flexner in 1910 for medical schools is characterized by a 4-year curricular education period with a bimodal approach in basic sciences and clinical subjects. Its use has been widespread around the world, and, thanks to certain adaptations, it has managed to last until today. This is despite its limitations, such as giving little importance to the primary care approach in undergraduate education [5–8]. Regardless of the teaching model used in academic training, the integration of radiology in the curriculum is indisputable. However, according to Kourdioukova et al. (2011), some aspects of the academic curriculum lack general consensus, such as the necessary number of hours of theoretical and practical classes to obtain a competent level of training upon completion of a medical degree [2,9]. In this context, due to the multifactorial nature of the teaching-learning process, various strategies have been proposed to improve students’ academic results, especially extracurricular activities such as elective courses and complementary readings [10,11].

The inappropriate prescription of radiologic studies by medical doctors represents a high cost for health systems and can be attributed to the lack of knowledge of their appropriate indications [12,13]. Moreover, exposure to radiation has harmful dose-dependent effects on health, such as the increase in the risk of certain types of cancer [14–16]. Nonetheless, previous studies have identified insufficient levels of knowledge in medical doctors and medical students regarding the risks of radiation exposure and the necessary protection measures to mitigate them [17–19]. Even when dealing with the clinical efficacy of imaging tests, medical students have shown poor knowledge [20].

Researchers who have evaluated the attitudes of medical students regarding their training in radiology have attributed students’ lack of knowledge of the subject to deficiencies in medical education. In this context, in 2012, Bhogal P. et. al highlighted the importance of academic training and argue that medical students during their medical degree must achieve a complete understanding of the available imaging techniques, including their basics of physics, interpretation, limitations and risks [2,21].

To the extent of our knowledge, no previous studies have described the curricular characteristics of radiology teaching along with the level of knowledge of this subject in medical students.

The aim of this study was to describe the level of knowledge and the curricular characteristics regarding the teaching of the subject of radiology in medical students in their final years (fifth and sixth years) of a medical degree from seven Latin American countries (Bolivia, Brazil, Colombia, Ecuador, Mexico, Paraguay, Peru).

## Materials and methods

### Hypothesis

This study was developed from testing strategy based on differences among groups. Our H0 was: No differences between countries in terms of knowledge about radiology. The H1 was: There are differences in terms of level of knowledge about radiology among countries.

### Ethics approval and consent to participate

This research used only anonymized information. In addition, its development was approved by the Research Ethics Committee of the Hospital General San Francisco de Quito (CEISH-HGSF), Quito, Ecuador, under the code: ‘CEISH-HGSF-2022–0014’.

### Study design

A descriptive, cross-sectional, multicenter study was performed using an online questionnaire.

### Setting and participants

An online survey was conducted between October 2019 and February 2020, in medical students from universities located in seven Latin American countries: Bolivia, Brazil, Colombia, Ecuador, Mexico, Paraguay, and Peru.

The participants were students legally enrolled in their finale two years of their degree: fifth year (year prior to the medical rotating internship) and sixth year (rotating internship year) who had already undertaken the subject of radiology as the main or complementary course of medicine at public or private universities in seven Latin American countries. For the selection of the participants and participating universities, a non-probabilistic sampling method was used for the convenience of the researchers. Thus, participants were recruited from universities described in the Additional file 1.

### Data measurement and questionnaire

The research team designed a structured questionnaire, to assess the level of knowledge of medical students about Radiology the researchers summarized the grids of their countries, chose related topics and formulated questions related to the physical basis of radiological tests, the most frequently performed radiological examinations, and risks of radiological examinations for the healthcare team and patients, based on the essential skills standards for medical students proposed by the Royal College of Radiologists (RCR) of the United Kingdom in 2017 [22], and the curricular characteristics of radiology in medical schools.

Prior to the study, a pilot study was carried out on 20 final-year medical students from the Universidad Central del Ecuador with the objective of identifying comprehension difficulties or errors within the structure of the questionnaire. After editing some questions due to errors detected in the piloting, a 40-item questionnaire was created in Spanish and revised and validated by three experts in Radiology and teaching. In addition, the questionnaire was translated into the Portuguese language by one of the Brazilian native researchers and validated by a Brazilian native expert in radiology to evaluate Brazilian medical students. Additionally, an English version of the questionnaire was designed to be displayed in this report (see Additional file 2).

The final version of the online research questionnaire was made up of three sections:

The first section included five questions about demographic variables such as: sex, age, year of study, country of residence and type of education (public or private).

The Second section was composed of 15 questions assessing the curricular characteristics of the teaching of Radiology in the participants’ medical schools, including: 1) the student perception of the importance of the radiology subject in academic training; 2) their perception about their level of radiology knowledge at the time of the study; 3) the semester/year of university education considered optimal to undertake the subject; 4) curricular characteristics of the subject as: percentage of the radiology syllabus (the planning tool for a subject) completed, percentage of radiology classes received by the participants based on the number of scheduled classes, teaching methodology, preferred bibliographic sources, extracurricular training activities in radiology and gaining radiology knowledge during their practical class hours in other medical specialties.

The third section included 20 questions consisted of 10 true/false questions and 10 multiple choice questions, which measured the level of radiology knowledge based on the following aspects: general knowledge of the most frequent radiologic studies, basics of physics of radiologic studies, and awareness of the risks of exposure to radiologic studies.

Data collection was carried out using the free access web tool ‘Google Forms’, in which a unique questionnaire was designed for each of the seven countries. Participants accessed the questionnaire through a link that was shared by the researchers through social network dissemination groups (Facebook and WhatsApp). In the initial section of all questionnaires, a brief explanation of the purpose of the study was presented, as well as a statement on the confidential handling of the data. In all cases, informed consent was obtained from the participants; likewise, the questionnaires collected online were anonymous and no personal information was requested in none of them.

### Bias

The biases to which the information collected was exposed were dealt with at different stages of the data collection and management process.

Initially, to deal with duplicate response bias, using the properties of the ‘Google Forms’ tool, the number of responses for the questionnaires was limited to 1 response for each IP device. In addition, during the completion of the surveys, in the case of the questions intended to evaluate aspects of the curricular characteristics of the radiology course (e.g., percentage of the syllabus completed, or percentage of lectures received), an explanation was added to each question related to the meaning of the variables being studied to avoid confusion among respondents. Regarding the ‘percentage of the syllabus completed’ variable, students were asked to optionally select the option ‘I did not receive any information about the radiology syllabus’, stated in the manuscript as ‘No information’.

Subsequently, to reduce bias, the researchers who analyzed the results did so independently and, if they found any errors or contradictions, they discussed them as a group with all the members of the research team to select the answers that were accepted as valid and included in the results of this research.

### Study size

The required sample size was calculated using the following equation, designed to calculate samples in infinite or unknown populations [23]:

$$n=\frac{Z^{2}\cdot \left(p\cdot q\right)}{e^{2}}$$

Starting from a confidence level of 95% (Z = 95%), margin of error of 7% (e = 7%) and an expected positive (p) and negative (q) distribution of 50%, a minimum of *n* = 196 completed questionnaires were obtained for each participating country.

### Data management

Within the demographic variables, the type of education was classified as public or private according to the source of funding of the University [24]. The year of study was classified as: fifth year and sixth year (for all participating countries the medical career lasts 12 semesters or 6 years; the sixth year represents the internship year for the universities of all countries except Brazil, where the internship rotation takes place during the 5th and 6th year of the career) of the medical degree. To assess the participants’ perceptions of radiology, the questions about the level of importance and the perception of the level of knowledge required single-choice answers. This was also the case with questions regarding the variables pertaining to the percentage of syllabus completed and the percentage of classes received. Permission to select more than one answer per question was granted in questions regarding the optimal moment to study the subject, teaching methodology, bibliographical sources, extracurricular activities, and the presence during the performance of radiological examinations in practical classes. Therefore, the total data collected for each question was greater than the total number of participants (*n* = 1514).

The level of knowledge in radiology was measured using the 10-point decimal type numerical grading scale [25], assigning a value of 0.5 points for each question answered correctly, while each incorrect question was assigned a value of 0.0 points (incorrect answers did not subtract points). Thus, the maximum score a participant could obtain was 10.0 points, and the minimum 0.0 points. In addition, the knowledge level qualification was stratified into three groups:

1. General knowledge of frequent radiologic studies, with 10 questions, allowing a maximum score of 5.0 points.
2. Basics of physics of radiologic techniques with five questions that allow a maximum score of 2.5 points.
3. Risks due to exposure to radiologic studies, with five questions that allow a maximum score of 2.5 points.

### Statistical methods

The descriptive analysis of the qualitative variables was carried out by evaluating frequencies and percentages. For quantitative variables, measures of the central tendency (mean) and dispersions (standard deviation) were analyzed.

To search for relationships of association between the variable’s ‘sex’, ‘year’, ‘education type’, ‘teaching methodology’, ‘information source’, and ‘be present during practical class’, with the knowledge level score the Student’s T-test was used. While searching for relationships of association between the variable’s ‘country’ ‘importance level’, ‘perception of knowledge level’, ‘percentage of the syllabus completed’, ‘percentage of classes attended as a function of classes programmed’ and ‘extracurricular activities’ with the knowledge level score, regression analysis of variables was performed, using the variable ‘knowledge level’ as the dependent variable, followed by a one-way ANOVA analysis for regressions with p-values<0.05. For all association analyses, a Bonferroni correction was developed to determine the p values accepted as statistically significant. Results analysis was carried out in the IBM SPSS version 24.0 software.

## Results

### Demographic characteristics

A total of 1,514 questionnaires were completed by medical students from Latin America. The country with the largest number of participants was Ecuador 16.4% (*n* = 248), but all countries had a similar sample size (*n* > 200). The 68.1% (*n* = 1031) were students under 24 years of age; 57.8% were women; 65.7% were enrolled in fifth year (the last year prior to internship); and 52.2% (*n* = 790) were from public universities (Table 1).

**Table 1.** Demographic characteristics and perceptions of Latin America medicine students.

		Country n (%)								Total	
			Bolivia	Brazil	Colombia	Ecuador	Mexico	Paraguay	Peru	n	%
Participants			206 (13.6)	209 (13.7)	231 (15.3)	248 (16.4)	217 (14.3)	200 (13.2)	203 (13.4)	1514	100.0
Age (years)	18–24		136 (13.2)	116 (11.3)	186 (18.0)	167 (16.2)	210 (20.4)	115 (11.2)	101 (9.8)	1031	68.1
	>24		70 (14.5)	93 (19.3)	45 (9.3)	81 (16.8)	7 (1.4)	85 (17.6)	102 (21.1)	483	31.9
Gender	Male		82 (12.8)	90 (14.1)	100 (15.7)	102 (16)	95 (14.9)	86 (13.5)	84 (13.2)	639	42.2
	Female		124 (14.2)	119 (13.6)	131 (15.0)	146 (16.7)	122 (13.9)	114 (13.0)	119 (13.6)	875	57.8
Year	Fifth year		142 (14.3)	110 (11.1)	199 (20.0)	114 (11.5)	201 (20.2)	150 (15.1)	79 (7.9)	995	65.7
	Sixth year		64 (12.3)	99 (19.1)	32 (6.2)	134 (25.8)	16 (3.1)	50 (9.6)	124 (23.9)	519	34.3
Education type	Public		27 (3.4)	199 (25.2)	117 (14.8)	185 (23.4)	195 (24.7)	32 (4.1)	35 (4.4)	790	52.2
	Private		179 (24.7)	10 (1.4)	114 (15.8)	63 (8.7)	22 (3.0)	168 (23.2)	168 (23.2)	724	47.8
Perception											
Importance level	Very Important		128 (15.3)	176 (21)	166 (19.8)	130 (15.5)	149 (17.8)	112 (13.3)	32 (3.8)	839	59.0
	Important		62 (12.5)	33 (6.7)	52 (10.5)	59 (11.9)	62 (12.5)	77 (15.5)	151 (30.4)	496	32.8
	Moderate		13 (11.9)	0 (0.0)	8 (7.3)	55 (50.5)	6 (5.5)	10 (0.9)	17 (15.6)	109	7.2
	Slightly important		2 (15.4)	0 (0.0)	5 (38.5)	4 (30.8)	0 (0.0)	0 (0.0)	2 (15.4)	13	0.9
	Unimportant		1 (33.3)	0 (0.0)	0 (0.0)	0 (0.0)	0 (0.0)	1 (33.3)	1 (33.3)	3	0.2
Perception of knowledge level	Very high		21 (25.3)	7 (8.4)	14 (16.9)	6 (7.2)	2 (2.4)	25 (30.1)	8 (9.6)	83	5.5
	High		82 (18.0)	54 (11.9)	61 (13.4)	42 (9.2)	44 (9.7)	116 (25.5)	56 (12.3)	455	30.1
	Regular		82 (11.2)	117 (16.0)	119 (16.3)	124 (17.0)	121 (16.6)	37 (5.1)	130 (17.8)	730	48.2
	Deficient		10 (5.0)	30 (15.1)	26 (13.1)	65 (32.7)	44 (22.1)	18 (9.0)	6 (3.0)	199	13.1
	Bad		11 (23.4)	1 (2.1)	11 (23.4)	11 (23.4)	6 (12.8)	4 (8.5)	3 (6.4)	47	3.1
Optimal time during the university degree to study the course	Along with basic sciences		36 (14.9)	20 (8.3)	54 (22.3)	63 (26.0)	47 (19.4)	14 (5.8)	8 (3.3)	242	9.4
	Along with preclinical subjects		86 (13.6)	183 (28.9)	75 (11.9)	53 (8.4)	59 (9.3)	61 (9.7)	115 (18.2)	632	24.4
	Along with clinical subjects		114 (11.2)	143 (14.1)	149 (14.7)	202 (19.9)	150 (14.8)	159 (15.6)	99 (9.7)	1016	39.3
	Along with surgical subjects		64 (9.2)	143 (20.6)	92 (13.2)	118 (17.0)	58 (8.3)	94 (13.5)	127 (18.2)	696	26.9

Table 1 displays the demographic characteristics of the participants by country, and the perceptions of medical students regarding Radiology.

### Students’ perceptions

Most medical students believe that radiology is very important (59%) or important (32.8%) to the academic training of the general practitioner; less than 1.0% believe the subject is not important. On the other hand, only 5.5% (*n* = 83) believe to have a very high level of knowledge in radiology, while 48.2% consider their knowledge level as regular. Of note, Paraguayan students had the best self-rating index regarding their Radiology knowledge (Table 1).

### Academic training characteristics

With respect to the level programmed to be taught, only 23.3% of students stated that between 75% and 94% of the contents proposed in their Radiology syllabus were fulfilled. The students who mostly claimed not to have received any information were Colombians (39.3%) and Paraguayans (25.5%). On the other hand, 17.6% claimed to have received less than 50% of scheduled classes, especially Colombian (44.7%) and Ecuadorian (21.8%) students. The most used teaching methodology was image analysis (32.1%). Regarding the most used bibliographic sources, books (29.0%) and professors’ opinions (26.3%) were the most cited ones. Moreover, 51.3% of the students said not to have participated in any extracurricular activity in Radiology. During their practical activities in in health centers, the students were more frequently present during the conduction of exams of lower complexity compared to those of higher complexity (Table 2).

**Table 2.** Radiology curricular characteristics of Latin America medicine students.

		Country n (%)							Total	
		Bolivia	Brazil	Colombia	Ecuador	Mexico	Paraguay	Peru		
	Respondents	n = 206	n = 209	n = 231	n = 248	n = 217	n = 200	n = 203	n	(%)
**Class characteristics**										
Percentage of the syllabus completed	95% − 100%	13 (5.1)	148 (58.3)	7 (2.8)	24 (9.4)	44 (17.3)	11 (4.3)	7 (2.8)	254	17.3
	75% − 94%	60 (17.5)	32 (9.4)	32 (9.4)	40 (11.7)	89 (26.0)	72 (21.1)	17 (5.0)	342	23.3
	50% − 74%	62 (18.6)	5 (1.5)	13 (3.9)	55 (16.5)	56 (16.8)	13 (3.9)	129 (38.7)	333	22.6
	Less 50%	22 (14.3)	2 (1.3)	26 (16.9)	42 (27.3)	17 (11.0)	5 (3.2)	40 (26.0)	154	10.5
	No information	49 (12.6)	22 (5.7)	153 (39.4)	44 (11.3)	11 (2.8)	99 (25.5)	10 (2.6)	388	26.4
Percentage of classes attended as a function of classes programmed	95% − 100%	58 (13.4)	143 (33.1)	34 (7.9)	32 (7.4)	73 (16.9)	79 (18.3)	13 (3.0)	432	29.6
	75% − 94%	71 (16.2)	57 (13.0	44 (10.0)	60 (13.7)	93 (21.2)	93 (21.2)	20 (4.6)	438	30.0
	50% − 74%	59 (17.6)	2 (0.6)	26 (8.7)	57 (17.0)	33 (9.9)	13 (3.9)	142 (42.4)	335	22.9
	Less 50%	18 (7.0)	7 (2.7)	115 (44.7)	56 (21.8)	18 (7.0)	15 (5.8)	28 (10.9)	257	17.6
Teaching methodology	Presentations conducted by students	52 (9.0)	64 (11.1)	86 (14.9)	188 (32.9)	100 (17.3)	16 (2.8)	71 (12.3)	577	22.2
	Clinical case analysis	54 (11.0)	98 (20)	70 (14.3)	43 (8.8)	31 (6.31)	53 (10.8)	142 (28.9)	491	18.9
	Images analysis	122 (14.6)	144 (17.3)	88 (10.6)	87 (10.4)	117 (14)	135 (16.2)	141 (16.9)	834	32.1
	Lectures given by professor	98 (14.1)	119 (17.1)	34 (4.9)	59 (8.5)	89 (12.8)	148 (21.2)	150 (21.5)	697	26.8
Bibliography source	Scientific articles	75 (11.7)	76 (11.9)	127 (19.9)	149 (23.3)	135 (21.1)	45 (7)	86 (13.5)	639	23.5
	Books	120 (15.3)	84 (10.7)	72 (9.2)	100 (12.7)	150 (19.1)	137 (17.4)	123 (15.6)	786	29.0
	Internet	48 (8.3)	114 (19.8)	76 (13.2)	95 (16.5)	73 (12.7)	61 (10.6)	109 (18.9)	576	21.2
	Professor’s opinion	100 (14)	104 (14.6)	86 (12)	63 (8.8)	90 (12.6)	131 (18.3)	140 (19.6)	714	26.3
Extracurricular activities	Virtual courses	11 (10.5)	21 (20.0)	34 (32.4)	16 (15.2)	12 (11.4)	4 (3.8)	7 (6.7)	105	7.0
	Attendance-based courses	35 (25.2)	17 (12.2)	19 (13.7)	15 (10.8)	15 (10.8)	15 (10.8)	23 (16.5)	139	9.3
	Extra readings	42 (11.2)	28 (7.4)	42 (11.2)	39 (10.4)	55 (14.6)	53 (14.1)	117 (31.1)	376	25.1
	Supervised practices	33 (30.3)	6 (5.5)	12 (11.0)	11 (10.1)	12 (11.0)	18 (16.5)	17 (15.6)	109	7.3
	None	84 (11.0)	126 (16.4)	123 (16.0)	164 (21.4)	122 (15.9)	110 (14.3)	38 (5.0)	767	51.3
Be present during practical class	X-rays	170 (13.2)	173 (13.4)	203 (15.8)	213 (16.5)	190 (14.8)	157 (12.2)	182 (14.1)	1288	33.6
	Ultrasound	113 (10.5)	163 (15.2)	171 (16.0)	197 (18.4)	175 (16.3)	124 (11.6)	128 (12.0)	1071	27.9
	Computed tomography	96 (10.4)	165 (17.9)	159 (17.2)	178 (19.4)	118 (12.8)	99 (10.7)	107 (11.6)	922	24.0
	Magnetic resonance	56 (10.1)	102 (18.4)	84 (15.1)	93 (16.7)	60 (10.8)	49 (8.8)	112 (20.1)	556	14.5

### Radiology knowledge level

In our whole sample (*n* = 1514), the average overall knowledge score was 5.5 ± 1.3/10 points. The sub scores for general radiology knowledge, basics of physics and risks of imaging techniques were 2.8 ± 0.8/5 points, 1.6 ± 0.6/2.5 points, and 1.1 ± 0.5/2.5 points, respectively. The country with the highest score was Brazil (6.1 ± 1.0 points), and the lowest level of knowledge was Bolivia (4.8 ± 1.4 points) (*n* < 0.001). In relation to knowledge components, the country with the highest risk knowledge was Brazil (1.4 ± 0.4 points), for basics of physics Paraguay (1.9 ± 0.5 points), and for general radiologic knowledge Ecuador (3.0 ± 0.8) (Table 3).

**Table 3.** Relationship between radiology knowledge level and curricular characteristics in Latin American medical students.

Characteristics				Radiology knowledge level					
				General knowledge	Basis of physics	Risks	Total	Bonferroni correction	T-test P value
			n	Mean/5.0 points ± SD	Mean/2.5 points ± SD	Mean/2.5 points ± SD	Mean/10.0 points ± SD		
Country	Bolivia		206	2.5 ± 0.9	1.4 ± 0.7	0.9 ± 0.5	4.8 ± 1.4	0.007	< 0.001*
	Brazil		209	2.9 ± 0.7	1.8 ± 0.5	1.4 ± 0.4	6.1 ± 1.0		
	Colombia		231	2.6 ± 0.9	1.5 ± 0.7	1.1 ± 0.5	5.3 ± 1.5		
	Ecuador		248	3.0 ± 0.8	1.6 ± 0.6	1.1 ± 0.4	5.8 ± 1.3		
	México		217	2.8 ± 0.8	1.6 ± 0.5	1.1 ± 0.4	5.4 ± 1.1		
	Paraguay		200	2.9 ± 1.0	1.9 ± 0.5	1.2 ± 0.5	6.0 ± 1.4		
	Perú		203	2.8 ± 0.6	1.2 ± 0.5	1.0 ± 0.4	5.0 ± 1.0		
Sex	Male		639	2.8 ± 0.8	1.7 ± 0.6	1.1 ± 0.5	5.6 ± 1.4	0.025	0.015
	Female		875	2.8 ± 0.8	1.5 ± 0.6	1.1 ± 0.4	5.4 ± 1.3		
Year	Fifth year		995	2.8 ± 0.9	1.6 ± 0.6	1.1 ± 0.5	5.4 ± 1.4	0.025	0.023
	Sixth year		519	2.9 ± 0.7	1.6 ± 0.6	1.1 ± 0.5	5.6 ± 1.3		
Education type	Public		790	2.9 ± 0.8	1.7 ± 0.6	1.2 ± 0.4	5.7 ± 1.3	0.025	< 0.001
	Private		724	2.8 ± 0.8	1.5 ± 0.6	1.1 ± 0.5	5.3 ± 1.4		
Importance level	Very Important		893	2.8 ± 0.8	1.6 ± 0.6	1.1 ± 0.4	5.6 ± 1.3	0.01	< 0.001*
	Important		496	2.9 ± 0.8	1.5 ± 0.6	1.1 ± 0.5	5.5 ± 1.3		
	Moderate		109	2.7 ± 0.8	1.5 ± 0.7	1.2 ± 0.5	5.3 ± 1.4		
	Slightly important		13	2.2 ± 1.1	1.2 ± 0.8	1.1 ± 0.4	4.5 ± 2.0		
	Unimportant		3	2.2 ± 2.1	1.0 ± 0.9	1.0 ± 0.0	4.2 ± 2.8		
Perception of knowledge level	Very high		83	2.5 ± 0.9	1.6 ± 0.7	1.1 ± 0.5	5.2 ± 1.5	0.01	< 0.001*
	High		455	2.9 ± 0.9	1.7 ± 0.6	1.1 ± 0.5	5.7 ± 1.4		
	Regular		730	2.8 ± 0.7	1.6 ± 0.6	1.1 ± 0.5	5.5 ± 1.2		
	Deficient		199	2.7 ± 0.8	1.5 ± 0.6	1.1 ± 0.4	5.3 ± 1.3		
	Bad		47	2.6 ± 0.8	1.3 ± 0.7	1.1 ± 0.5	5.0 ± 1.4		
Percentage of the syllabus completed	95% − 100%		254	2.9 ± 0.8	1.8 ± 0.5	1.3 ± 0.4	6.0 ± 1.2	0.01	< 0.001*
	75% − 94%		342	2.9 ± 0.9	1.7 ± 0.5	1.2 ± 0.5	5.8 ± 1.4		
	50% − 74%		333	2.8 ± 0.7	1.4 ± 0.6	1.0 ± 0.4	5.2 ± 1.1		
	Less 50%		154	2.8 ± 0.8	1.4 ± 0.6	1.0 ± 0.4	5.2 ± 1.3		
	No information		431	2.7 ± 0.8	1.6 ± 0.7	1.1 ± 0.5	5.4 ± 1.4		
Percentage of classes attended as a function of classes programmed	95% − 100%		432	2.9 ± 0.8	1.8 ± 0.5	1.2 ± 0.4	5.9 ± 1.2	0.013	< 0.001*
	75% − 94%		438	2.8 ± 0.9	1.6 ± 0.6	1.1 ± 0.5	5.6 ± 1.4		
	50% − 74%		387	2.8 ± 0.7	1.4 ± 0.6	1.0 ± 0.4	5.2 ± 1.2		
	Less 50%		257	2.6 ± 0.8	1.4 ± 0.7	1.0 ± 0.4	5.1 ± 1.4		
Teaching methodology	Presentations conducted by students	Yes	577	2.8 ± 0.8	1.6 ± 0.6	1.1 ± 0.4	5.5 ± 1.3	0.025	0.886
		No	937	2.8 ± 0.8	1.6 ± 0.6	1.1 ± 0.5	5.5 ± 1.3		
	Clinical case analysis	Yes	491	2.8 ± 0.8	1.6 ± 0.6	1.1 ± 0.5	5.6 ± 1.3	0.025	0.162
		No	1023	2.8 ± 0.8	1.6 ± 0.6	1.1 ± 0.5	5.5 ± 1.3		
	Images analysis	Yes	834	2.9 ± 0.8	1.6 ± 0.6	1.2 ± 0.4	5.7 ± 1.2	0.025	< 0.001
		No	680	2.7 ± 0.9	1.5 ± 0.6	1.1 ± 0.5	5.3 ± 1.4		
	Master classes	Yes	697	2.9 ± 0.8	1.6 ± 0.6	1.1 ± 0.4	5.7 ± 1.2	0.025	< 0.001
		No	817	2.7 ± 0.8	1.5 ± 0.6	1.1 ± 0.5	5.4 ± 1.4		
Information source	Scientific articles	Yes	693	2.9 ± 0.8	1.6 ± 0.6	1.1 ± 0.4	5.6 ± 1.3	0.025	0.043
		No	821	2.8 ± 0.8	1.5 ± 0.6	1.1 ± 0.5	5.4 ± 1.4		
	Books	Yes	786	2.9 ± 0.8	1.6 ± 0.6	1.1 ± 0.5	5.6 ± 1.3	0.025	0.037
		No	728	2.8 ± 0.8	1.6 ± 0.6	1.1 ± 0.5	5.4 ± 1.3		
	Internet	Yes	576	2.9 ± 0.7	1.6 ± 0.6	1.1 ± 0.4	5.5 ± 1.2	0.025	0.341
		No	938	2.8 ± 0.9	1.6 ± 0.6	1.1 ± 0.5	5.5 ± 1.4		
	Professor’s opinion	Yes	714	2.8 ± 0.8	1.6 ± 0.6	1.1 ± 0.5	5.6 ± 1.3	0.025	0.152
		No	800	2.8 ± 0.8	1.6 ± 0.6	1.1 ± 0.5	5.5 ± 1.3		
Extracurricular activities	Virtual courses		105	2.8 ± 0.8	1.7 ± 0.6	1.2 ± 0.5	5.7 ± 1.3	0.01	0.825*
	To face courses		139	2.8 ± 0.8	1.6 ± 0.6	1.0 ± 0.4	5.4 ± 1.3		
	Extra readings		376	2.8 ± 0.8	1.5 ± 0.6	1.1 ± 0.4	5.4 ± 1.3		
	Supervised practices		109	2.7 ± 0.8	1.6 ± 0.7	1.1 ± 0.4	5.4 ± 1.2		
	None		767	2.8 ± 0.8	1.6 ± 0.6	1.1 ± 0.5	5.5 ± 1.4		
Be present during practical class	X-rays	Yes	1288	2.8 ± 0.8	1.6 ± 0.6	1.2 ± 0.5	5.6 ± 1.4	0.025	0.008
		No	226	2.7 ± 0.8	1.5 ± 0.6	1.1 ± 0.5	5.3 ± 1.4		
	Ultrasound	Yes	1071	2.9 ± 0.8	1.6 ± 0.6	1.1 ± 0.5	5.7 ± 1.3	0.025	< 0.001
		No	443	2.6 ± 0.9	1.4 ± 0.6	1.1 ± 0.5	5.2 ± 1.4		
	Computed tomography	Yes	922	2.8 ± 0.8	1.7 ± 0.6	1.2 ± 0.5	5.7 ± 1.3	0.025	< 0.001
		No	592	2.8 ± 0.9	1.5 ± 0.6	1.1 ± 0.5	5.4 ± 1.4		
	Magnetic resonance	Yes	556	2.9 ± 0.8	1.6 ± 0.6	1.2 ± 0.4	5.8 ± 1.3	0.025	0.002
		No	958	2.8 ± 0.9	1.5 ± 0.6	1.1 ± 0.5	5.4 ± 1.4		
Total score			1514	2.8 ± 0.8	1.6 ± 0.6	1.1 ± 0.5	5.5 ± 1.3		

SD: Standard deviation; *p-Value obtained from regression analysis and one-way ANOVA.

Male participants showed the highest knowledge level (5.6 ± 1.4) compared to women (*p* = 0.015). Likewise, students enrolled in sixth year showed a higher score (5.6 ± 1.3 points) compared to those from fifth year (*p* = 0.023), and students enrolled in public universities had a higher score relative to private universities (5.7 ± 1.3 points) (*p* < 0.001) (Table 3). The importance given to radiology by the participants showed a positive inclination toward the level of knowledge, those who considered radiology as a very important subject had the highest knowledge scores (5.6 ± 1.3 points) (*p* < 0.001). In addition, compliance with the syllabus, as well as compliance with the scheduled classes, showed positive effects on the level of knowledge with higher scores in the higher compliance percentages (*p* < 0.001) (Table 3). Regarding teaching methodology, overall, image analysis and master classes were the techniques with the highest levels of knowledge (5.7 ± 1.2 points), and when analyzing the techniques individually, image analysis and master classes showed significantly higher levels of knowledge compared to those who said they did not use them (*p* < 0.001). In terms of the information source, participants who claimed to use the Internet had the lowest knowledge, while in the individual analysis, students who used scientific articles and books had higher knowledge compared to those who did not (*p* < 0.05) (Table 3). Finally, the development of radiologic studies during clinical practice showed greater knowledge in studies of greater complexity, likewise, the presence of students in any radiologic study exposed higher levels of knowledge compared to not being present (*p* < 0.05) (Table 3).

Table 3 presents the level of radiology knowledge expressed as mean and standard deviation of Latin American students out of 10 points, stratified according to general knowledge out of 5.0 points, basics of physics of radiology techniques out of 2.5 points, and risks out of 2.5 points. In addition, the relationship between the overall knowledge level with the demographic and curricular variables.

## Discussion

**Figure 1.**
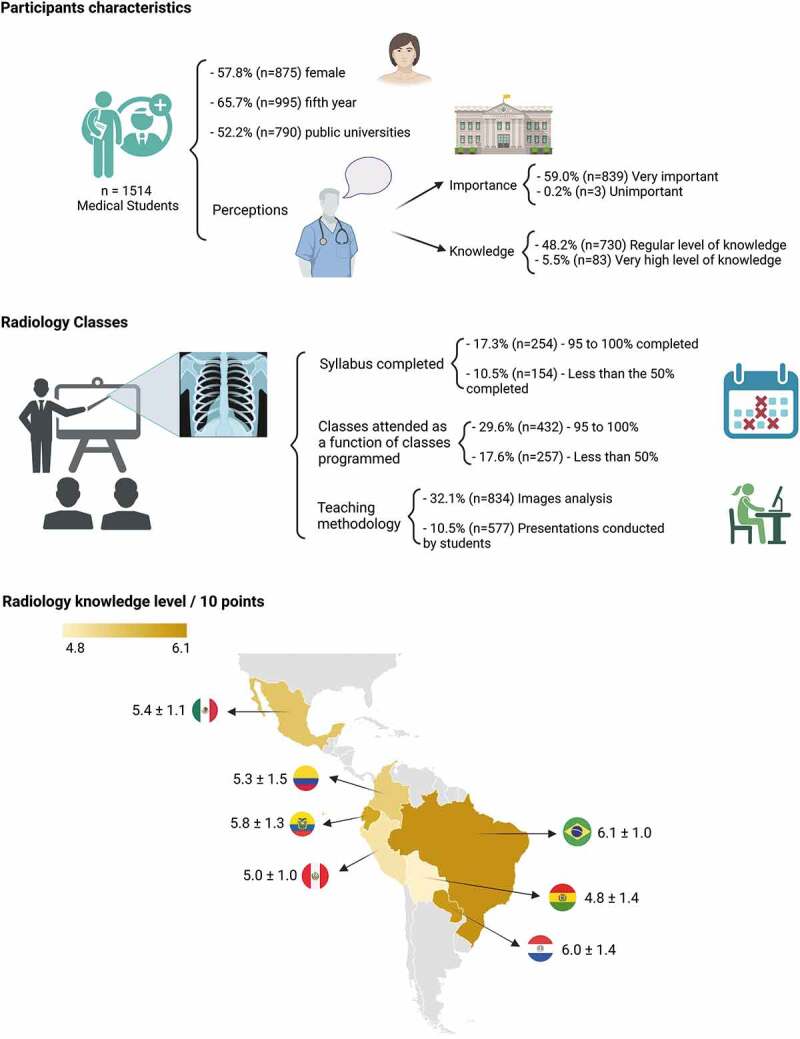
Main findings about the level of knowledge of radiology in Latin American medical students.

In addition to revealing the radiology knowledge level for medical students in their last years in several Latin American countries, the findings of this multicenter research allow for a better understanding of the teaching-learning process and the role played by curricular variables in this process, the main findings of these research are summarized in the Figure 1. The participants in our study showed a predominance of female respondents, probably justified because women have shown a greater predisposition to answer online surveys [26], or probably because it has been suggested that in recent years there is a higher percentage of female medical students, however, there are no official data in Latin America.

According to the type of university, although our participants had a similar overall distribution between public and private universities, in several countries there was a much higher frequency of participants from public universities, and students from public universities showed a significantly higher level of knowledge. We believe that these differences may be indirectly caused by the fact that several public universities in the Latin American region are more in demand than private universities. Another characteristic of our study is that the population chosen to participate in this research focused on students who were finishing their medical degree. This decision was taken since we consider that at this point in their training, they should have knowledge close to what is necessary for the development of their medical practice. What our findings showed is that the students who were attending the sixth year (participating in their medical internship) have significantly higher levels of knowledge compared to the group of fifth year. We believe that these differences make sense, due to the enormous load of academic theory and practice that the internship provides in medical degree training compared to the previous years. Accordingly, our research supports the results found by Leschied J. et al. who, with short elective courses in radiology, evidenced an improvement in the level of knowledge [27], as well as the report by Dawes et al. who demonstrated an improvement in the performance in the interpretation of images (radiological cases) after 26 weeks of clinical training [28]. It is important to note that there exists a relative heterogeneity in the ‘year of study’ variable, given that in Brazil, despite having the same degree duration, the last two years of a medical degree correspond to the internship, rather than just the final year, meaning that greater exposure to clinical radiology. We consider that the possible effects of this difference can be evidenced in our findings since the Brazilian participants obtained the highest knowledge scores, although more precise studies would be necessary in this regard.

It has been described that the perception of the student and teacher with respect to the importance of the material being studied, in the degree in the generation of student knowledge on the theme [29,30]. This research reveals a strong positive association between the perceived importance that the student perceives radiology to have in their degree and the level of knowledge that they achieve with respect to the same. This highlights a potential role of the teacher in emphasizing the relevance of a theme for student results and corroborates findings seen in previous studies carried out in medical students in the United Kingdom and the United States for whom radiology is also very important in their training [30,31]. On the other hand, despite the fact that our participants attach great importance to the subject, most of them claimed to have only a regular level of knowledge in radiology although being in the final stages of their medical degree, a discrepancy of opinions that has been noted in several studies [31,32].

We found alarming information about the quality of university education in our participants, an important part (56.4%) reported that the teachers responsible for teaching the classes complied with less than 75% of the topics that had been programmed in the academic radiology curriculum, Peru and Ecuador being the countries most affected by this characteristic; as well as in 40.5% of cases, the programmed classes were taught by the teachers in less than 75%, this deficiency affecting more the students of Peru and Colombia. In both cases, a positive association was observed between higher levels of knowledge and higher percentages of compliance with the curriculum (scheduled classes and syllabus). On the other hand, regarding to the methodology used by the teachers, a significant percentage of participants stated that they experienced the classes through presentations made by the students; this methodology obtained the lowest knowledge score. While the only methods that showed a significant positive effect on the level of knowledge were images analysis and master classes, we found that our findings are partially supported by the study of Nyhsen C. et al. who found that medical students from Newcastle, UK, rated case-based radiology teaching as the most effective, while student-led (self-directed) learning as the least effective [33].

This investigation sought to analyze learning scenarios close as possible to the reality of the teaching process. Accordingly, the study evaluated the relationship between academic teaching activities that are not included in the curricular documents (syllabus) or that are taught spontaneously in the teacher-student relationship. Such teaching approaches are referred to in the literature as part of a ‘hidden curriculum’ for which neither the educational institution nor the student is responsible [34]. What our research finds is that there is a strong relation between the participant presence during the performance of imaging techniques evaluated (X-ray, ultrasound, computer tomography and magnetic resonance) during a practical class that are not directly related to the radiology subject, and the level of knowledge that the participant gains. As is also to be expected, the presence of the students decreases as the radiological studies that are performed gain complexity (computed tomography and magnetic resonance). However, it was found that the students’ knowledge score increases with the complexity of the radiological study.

The overall level of knowledge of Latin American medical students in radiology is regular (5.5 ± 1.3/10.0 points). Taking as a point of ‘approval or acceptance’ a score≥7.0 points, demonstrating that the average level of knowledge could be categorized as ‘insufficient’. Also, a deficiency of knowledge was observed in all the categories studied: general knowledge about common radiological studies (mean of 2.8 ± 0.8/5.0 points), the fundamentals of the physics of radiological studies (mean of 1.6 ± 0.6/2.5 points) and the potential risks involved in radiological studies for the patient and healthcare personnel (mean of 1.1 ± 0.5/2.5 points). These findings are not new in the literature. Several research in medical students of various levels have stated that they have low levels of knowledge about indications for common medical imaging modalities, radiation doses, and risks associated with imaging exam agents that use ionizing radiation [18,21,35,36]. The findings of the present investigation, as well as others, suggest the existence of an important deficit of radiology knowledge during university training, which could extend to the professional life of the medical student. In particular, it could be considered that students with deficient levels of knowledge will become professionals with deficient knowledge for professional medical practice [17,37,38].

The deficiency of correlation between the importance that radiology has in modern medical practice and the lack of importance given to the same in the academic curricula of medical schools around the world is considered a significant concern [2,11]. Although there are multiple reports regarding deficiencies in the knowledge that students and professionals (medical doctors) have on this subject in the region [32,39–41],, efforts to improve the academic training of medical students in Latin America have only been demonstrated by Chilean academics who have proposed an academic curriculum for radiology that seeks to include all the knowledge that a general practitioner should have [42][]. While this is a significant achievement in Latin America, within other regions, the efforts are much greater, important examples include the Royal College of Radiologists in the United Kingdom, which produces routine guidelines of recommendations for the preparation in radiology of medicine students [22]. This approach is based on the findings that many authors assert having a good foundations in radiology are essential for medical practice and its capacity to result in more efficient practice by minimizing the number of unnecessary studies, reducing the risk of harmful effects in patients and ensuring a better use of financing sources [43].

The authors of the present study acknowledge that the teaching-learning process is far from being fully understood, however, that the objectives set out in this research were achieved. Specifically, we were able to confirm the low quality of knowledge that Latin American students have, as well as deficiencies that exist in the radiology teaching process as an important part of the medical degree training. In addition, the present investigation showed that the characteristics of academic formation are dependent on all the actors of the teaching-learning process, and that of the role of the teacher, which shows to be essential. The implication of this result calls for renewed attention to ensuring quality teaching, a petition made nearly two decades ago by Rogers L., but that has failed to be taken with adequate importance until these days [44].

## Limitations

This study has several limitations in the ability to form robust conclusions. An important limitation is that of selection bias, as the delegates of each country were forced to opt for a convenience sampling to obtain the necessary sample, contacting Universities that were within their reach to distribute the survey. Consequently, the results are not representative of the country population. Nevertheless, the effects of selection bias were reduced by including students from private and public universities. The self-report design also exposes the research to potential selection bias as students interested in radiology are more likely to have been willing to complete the questionnaire. Another limitation is that the evaluation system used to evaluate the level of knowledge of the students who participated was developed by the researchers for this study. To ensure, however, that the surveys did capture objective results, measures were proposed to validate the knowledge level evaluation instrument. In particular, the radiology experts were asked to approve that the questions be equally weighted. In addition, the assessment system used was taken from the decimal-type numerical grading scale that gives equal weighting to the answers to each question addressing separate the items.

Regarding the questionnaire, an important limitation is that the radiology knowledge assessment tool only included theoretical knowledge and did not include important practical aspects such as the ability to recognize anatomical elements, radiological patterns, identification of pathologies, or direct interpretation skills (using images), which is an approach for future research. The study was also limited by the possibility that the measurement of radiology knowledge outcomes could be biased, as there are no national guidelines described in the participating countries that end up with a common or specific range for radiology education, so the investigators developed the knowledge questions based on the precepts described by the Royal College of Radiologists on the radiology knowledge that the general practitioner should possess at the end of the training, and after that, we obtained the approval of radiology experts who assured that the questionnaire was intended to measure an elementary level of knowledge. We believe that these biases were adequately addressed, as the results showed similar distribution trends among the groups of participants in each country.

According to the training in radiology, a limitation was found because we could not evaluate the variables of the number of hours of the subject because no official documents detailing the number of hours or courses in radiology for the students of the participating countries are available. Another limitation is that school-level analyses could not be conducted because official information regarding the number of participants officially enrolled in each course was not provided to the researchers, however, the researchers managed to cover a sample of more than 200 participants per country to be considered representative.

Finally, to address the possibility of social acceptance bias that could be due to students fearing the lack of anonymity of the responses, researchers tried to reiterate to students the strict anonymity of the data collected and the importance of honesty when completing the instrument.

## Further research

Our work is the first of its kind, with a broad exploratory character on the teaching-learning process in medical students, a phenomenon of very complex characterization, as evidenced by the limitations reported in this manuscript. Nevertheless, the findings presented in this study represent a baseline study that may be useful for future explorations and comparisons.

## Conclusions

Latin American medical students in the final two years of their medical degree have an overall level of radiology knowledge considered only regular. Despite the multifactorial origin of the teaching-learning process, the curricular differences that characterize the academic training process such as a type of education (public or private), compliance with classes and academic programs, and teaching methodologies seem to have an influence on the level of knowledge that students achieve. The undoubted importance of equipping medical students to be qualified and efficient future practicing physicians should incline all actors in the educational system to strive to understand and improve the academic training of these students, not only in the radiology subject. This can be achieved through a cycle of continuous evaluation and improvement [43,44].

## Supplementary Material

Supplemental Material

## Data Availability

The entire data set is available by written request to the lead or corresponding author.
