# Supplementary material for: How much do Latin American medical students know about radiology? Latin-American multicenter cross-sectional study
Source: Med Educ Online. 2023 Feb 1;28(1):2173044. doi: 10.1080/10872981.2023.2173044 (PMC9897776; doi:10.1080/10872981.2023.2173044)
Supplement: Supplemental Material [file ZMEO_A_2173044_SM1001.zip › Supplementary/Additional file 1 Universities participating.docx]

Additional file 1.

The participants of the study were recruited from the following universities:

- Bolivia: Universidad Unifranz, Universidad Autónoma Gabriel René Moreno, Universidad Mayor de San Andrés and Universidad Mayor de San Simón.
- Brazil: Universidad Federal de Minas Gerais and Pontificia Universidade Católica de Minas Gerais.
- Colombia: Universidad del Quindío, Universidad de Manizales and Fundación Universitaria Autónoma de las Américas.
- Ecuador: Universidad Central del Ecuador, Pontificia Universidad Católica del Ecuador and Universidad de Las Américas.
- Mexico: Universidad de Guadalajara, Universidad Lamar and Universidad Autónoma del Estado de México.
- Paraguay: Universidad Católica Nuestra Señora de la Asunción, Universidad Nacional de Asunción and Universidad del Pacífico.
- Peru: Universidad Privada San Juan Bautista, Universidad San Martín de Porres and Universidad Mayor de San Marcos.
