# Supplementary material for: How much do Latin American medical students know about radiology? Latin-American multicenter cross-sectional study
Source: Med Educ Online. 2023 Feb 1;28(1):2173044. doi: 10.1080/10872981.2023.2173044 (PMC9897776; doi:10.1080/10872981.2023.2173044)
Supplement: Supplemental Material [file ZMEO_A_2173044_SM1001.zip › Supplementary/Additional file 2 Questionnaire.docx]

**“Radiology knowledge level of Latin American medical students"**

1. Sex:
   1. Male
   2. Female
2. Years of age:

_______ years.

1. University:

__________________

1. What year (medical degree) are you currently studying?
   1. Fifth year (nineth or tenth semester).
   2. Sixth year (Internship).
2. What is the method most used by your teacher during your radiology classes? You can choose more than one answer.
   1. Presentations conducted by students
   2. Clinical case analysis
   3. Images analysis
   4. Lectures given by professor
   5. Other
3. What is your preferred source of radiology information? You can choose more than one answer.
   1. scientific articles
   2. Books
   3. Web pages
   4. Professor's opinion
   5. Other
4. Do you participate, or have you participated in extracurricular activities that reinforce your learning in radiology subject?
   1. Virtual courses
   2. Attendance-based courses
   3. Extra readings
   4. Supervised practices
   5. Other
   6. None
5. What do you think is the best time to take the subject of radiology? You can choose more than one answer.
   1. Along with basic sciences (anatomy, embryology, histology)
   2. Along with preclinical subjects (anatomopathology, immunology)
   3. Along with clinical subjects (medical clinic: cardiology, endocrinology, neurology, etc.)
   4. Along with surgical subjects (general surgery, traumatology, etc.)
6. How important is the subject of imaging during clinical practice for you?
   1. Very important
   2. Important
   3. Moderate
   4. Slightly important
   5. Unimportant
7. What level of knowledge do you think you have about the subject of radiology?
   1. Very high
   2. High
   3. Regular
   4. Deficient
   5. Bad
8. Have you been present (as examiner or examiner's assistant) during the analysis of the radiological studies below?

|  | **Study** | **Never** | **Yes** |
| --- | --- | --- | --- |
| 1. | Nuclear magnetic resonance |  |  |
| 2. | Computed Axial Tomography |  |  |
| 3. | Radiography (X-ray) |  |  |
| 4. | Ultrasonography |  |  |

1. What percentage of radiology syllabus content was completed during your training?
   1. 95% - 100%
   2. 75% - 94%
   3. 50% - 74%
   4. Less than 50%
   5. I did not receive any information about the syllabus.
2. What was the average percentage of theoretical and practical classes attended during your radiology training?
   1. 95% - 100%
   2. 75% - 94%
   3. 50% - 74%
   4. Less than 50%

**Radiology Knowledge**

**According to your knowledge about radiological studies. Answer the following questions by selecting "true", "false" or "don't know" by marking an (X) in the correct space.**

|  | **Question** | **True** | **False** | **Don’t know** |
| --- | --- | --- | --- | --- |
| 1. | The patient must have a full bladder to perform a transabdominal pelvic ultrasound. |  |  |  |
| 2. | During a CT scan: the patient advances through the scanner on a moving platform, instead of the scanner moving over the patient. |  |  |  |
| 3. | It is usually difficult for a patient to remain still (without movement) for the time required to perform the CT scan. |  |  |  |
| 4 | MRI scans usually take longer than CT imaging studies of the same body area. |  |  |  |
| 5. | Patients often complain of muscle pain after MR. |  |  |  |
| 6. | The radiation exposure suffered by a patient for an abdominal X-ray is greater than for a plain chest X-ray. |  |  |  |
| 7. | In a pregnant woman undergoing a chest X-ray. There is a significant (dangerous) radiation exposure to the fetus. |  |  |  |
| 8. | Patients undergoing pelvic ultrasound are exposed to radiation. |  |  |  |
| 9. | MR is contraindicated for patients who have had intracranial vascular clipping because of recent aneurysm repair. |  |  |  |
| 10. | The standard chest X-ray is taken in the anterior-posterior direction. |  |  |  |

*CT: Computed Axial Tomography, MR: magnetic resonance.

**In the following questions. Select the literal of the option that you consider correct.**

1. **What are the risks of exposure to magnetic resonance?**
   1. None
   2. Exposure to ionizing radiation
   3. Malfunction of pacemakers and heart valve prostheses
   4. Opening of cerebral aneurysm clips
   5. Injury from metallic projectiles
   6. Nephrogenic systemic fibrosis with contrast
2. **When should patients be informed of the risks of the radiological studies they are going to receive?**
   1. Always
   2. Almost always
   3. Sometimes
   4. Never
3. **In an adult patient with effacement of the lateral diaphragmatic cost angle. There is at least pleural effusion of:**
   1. 50ml
   2. 25ml
   3. 100ml
   4. 150ml
   5. None
4. **Before interpreting a chest X-ray, which parameters indicate the development of the study under an adequate technique?**
   1. Positioning and centering
   2. Inspiration degree
   3. Penetration degree
   4. Patient identification
   5. All are correct
   6. A and B are right
5. **The inspiration degree on a good quality chest X-ray can be checked by:**
   1. With clavicles symmetry
   2. Absence of foreign bodies
   3. Counting to the 7th/8th posterior costal arches
   4. Dorsal vertebral bodies are seen to be clear
   5. None
   6. All are correct
6. **What structures of the cardiac silhouette can be seen on a normal chest X-ray?**
   1. Right ventricle
   2. Left atrium
   3. Left ventricle
   4. Right atrium
   5. C and D are right
   6. None
7. **In which case should you ask for an expiratory chest X-ray?**
   1. Pneumothorax
   2. Pneumomediastinum
   3. Left pleural effusion
   4. None
8. **Which of the following radiological studies emits radiation?**
   1. X-rays
   2. Ultrasonography
   3. Computed Axial Tomography
   4. Nuclear magnetic resonance
   5. A and C are correct
   6. None
9. **Which of the following is the best measure of protection against radiation emitted by radiological studies?**
   1. Reduce the milliamperage of the plate.
   2. Avoid unnecessary examinations of patients
   3. Reduce the kilovoltage of projections
   4. Reduce radiation exposure time
   5. None
10. **The radiologic sign of "acoustic shadow" is characteristic of the following pathology. Select an answer:**
    1. Fatty liver
    2. Polycystic ovarian syndrome
    3. Lithiasis
    4. Lipoma
    5. None
